# Optimisation of bioaerosol sampling using an ultralight aircraft: A novel approach in determining the 3-D atmospheric biodiversity

**DOI:** 10.1016/j.heliyon.2024.e38924

**Published:** 2024-10-12

**Authors:** Maria P. Plaza, Athanasios Charalampopoulos, Vivien Leier-Wirtz, Pia Viuf Ørby, Mathilde Kloster, Michael Dines Christiansen, Claudia Traidl-Hoffmann, Athanasios Damialis, Ulrich Gosewinkel

**Affiliations:** a Institute for Environmental Medicine and Integrative Health, Environmental Medicine, Faculty of Medicine, University of Augsburg and University Hospital Augsburg, Augsburg, Germany; b Institute of Environmental Medicine, Helmholtz Munich – German Research Center for Environmental Health, Augsburg, Germany; c Department of Ecology, School of Biology, Faculty of Sciences, Aristotle University of Thessaloniki, Thessaloniki, Greece; d Department of Environmental Science, Aarhus University, Roskilde, Denmark; e Big Data Centre for Environment and Health (BERTHA), Aarhus University, Aarhus, Denmark; f Astma-Allergi Danmark, Roskilde, Denmark; g Dines Avitech Aps, Havdrup, Denmark; h Christine Kühne-Center for Allergy Research and Education (CK-CARE) Davos-Augsburg-Bonn-St Gallen-Zürich, St Gallen, Switzerland

**Keywords:** Aerobiology, Bioaerosol monitoring, Fungal spores, Air biome, Airborne sampling, Isokinetic impactor, Pollen

## Abstract

Bioaerosols, such as pollen and fungal spores, are routinely monitored for agricultural, medical or urban greening practices, but sampling methodology is largely relying on techniques more than half a century old. Moreover, biomonitoring campaigns often take place in urban environments, although sources can be located outside cities’ borders with ampler vegetation. Therefore, the question arises whether we are accurately picturing the biodiversity and abundance of regional bioaerosols and whether those locally detected might derive from long-distance transport, horizontally or vertically. To answer the above, we used novel, mobile monitoring devices, and aerial measurement units, like aircrafts, so as to explore bioaerosol concentrations at a variety of altitudes.

An ultralight aircraft was equipped with a sampling device for bioaerosols. The device consisted of duplicate isokinetic impactors that match the physical functioning and the microscopic quantification method of the widely used ground-based Hirst-type impactors. Isokinetic airflow was realized by adjusting the air flux at the impactors’ inlet to the airspeed of the aircraft. Three campaigns were made, where the comparability, efficiency and accuracy of different sampling devices were determined, namely of the abovementioned impactor, and of the mobile conventional Hirst-type pollen sampler. The campaigns involved measurements from ground level (0 m altitude) up to 900 m (above ground level (agl)) via flights.

Our results showed that aircraft-based airborne pollen concentration measurements were consistently higher than those of all other devices, regardless of the altitude and sampling time. It is noteworthy that the pollen concentration exceeded 500 pollen grains/m^3^ at >900 m of altitude, this concentration being 1.77 times higher than that simultaneously measured at ground level. Likewise, the diversity of pollen was also higher at higher altitude.

Our results indicate the usability and superiority of small aircraft and high-flow impactors for research, achieving higher biodiversity and abundance over a shorter sampling interval compared to conventional volumetric techniques. Higher pollen amounts at higher altitudes also point at the necessity to monitor bioaerosols across the vertical dimension, especially in densely populated areas and high-traffic air space.

## 1 Introduction

The development of airborne bioaerosol monitoring has evolved in close relation to medical research, as airborne pollen and fungal spores constitute one of the main causes of respiratory allergies worldwide [1,2]. This situation is expected to worsen due to climate change [3,4], exacerbating the synergistic effects of pollutants and respiratory infections on asthma [5] for most allergenic taxa, with earlier pollen seasons [[6], [7], [8]], as well as higher pollen production and intensity [9]. Accordingly for fungal spores, there is little evidence on their behavioural patterns and trends due to the effects of climate change. Nonetheless, it is highly expected that infectious and non-infectious diseases in humans will increase, along with an increase of the cases of pathogens for animals, plants and food, due to their adaptability to the new conditions and also the geographical expansion of their distribution [10,11].

Monitoring networks are generally established in or near urban areas [12], at ground level or at a maximum height of 30 m [13] to collect the different pollen taxa representative of the broader environment, and have so far facilitated our understanding and prediction of pollen seasons [14,15]. However, airborne pollen is also the fingerprint of vegetation types [16]. It reflects phenological, reproductive and dispersal responses of plants as well as changes in land use and biodiversity representing large areas. Several studies, using manned and unmanned aircraft, hot air balloons and drones [[17], [18], [19], [20], [21]], have shown that pollen could be transported by air masses moving at an altitude of >1000 m above sea level [20,22] and travel long distances of >500 km from their source, under specific climatic conditions [23]. The same type of information, source-wise, is difficult to obtain for fungal spores, as they use multiple substrates for growth and their ecological requirements are characterised by high variability, both spatially and seasonally [24]. Adding to this, the cosmopolitan distribution of some fungi and subsequently their spores can make it difficult to study and estimate their long-distance transport patterns.

Bioaerosol sampling is therefore an important factor that can help us understand in more detail the effect of recent and future global environmental changes [25] on plant and fungal dynamics, their interactions with the environment, and ultimately on human health. Therefore, studies encompassing both environmental factors and bioaerosols need to be conducted in both urban and rural settings independent of altitude, and novel multidisciplinary approaches are needed to more accurately record and thus predict their atmospheric abundance. The identification and quantification of bioaerosols is essential to address related hazards [26] and to establish appropriate exposure thresholds. They have traditionally been monitored using manual samplers, such as the Hirst-type pollen and spore trap [27] or the Rotorod [28], and aerobiological networks have been established at national and international level [12] with standardised sampling methods [29].

In recent years only, automated systems using different particle recognition techniques have been incorporated to provide real-time data [[30], [31], [32], [33], [34], [35], [36], [37]]. Technologies and methods are evolving rapidly and new instruments are being developed continuously. To evaluate all currently available commercial instruments and research prototypes, an international intercomparison was organised under the auspices of the EUMETNET AutoPollen Programme and the ADOPT COST Action (CA18226). However, changes in bioaerosol diversity and abundance at the microscale level or in altitudinal profile have not yet been investigated in detail. Detection and three-dimensional modelling of aeroallergen transport are in its infancy, and generalised and comprehensive 3D modelling of bioaerosol concentration and transport is still difficult. To add up to the complexity, a limiting factor for such observations is also the scarcity of low-cost and mobile instruments and the penetration of new related techniques at the frontiers of atmospheric biology research. Multidisciplinary approaches are essential for a better understanding and more accurate prediction of bioaerosol transport patterns.

Therefore, the main goal of this study was to test a newly developed and novel method for the 3-dimensional atmospheric detection and monitoring of bioaerosols by use of an ultralight manned aircraft, as well as the standardization and applicability of the techniques acquired at the operational and real-life level. Thus, associated warning alerts could potentially be developed and more efficiently disseminated for the clinically relevant human exposure to aeroallergens. To validate the operability and comparability of such a novel detection technique, a measurement campaign was implemented on three different days, with several instruments and at various altitudes.

## 2 Material and methods

### 2.1 Study area

Air samples were collected at two locations in Denmark during June 2021: Roskilde-Risø and Copenhagen-Ryparken. The first two measurement series were done at Roskilde-Risø on a flat rooftop 4 m above ground level, utilizing a well-described sampling station for routine air pollution monitoring (N55.69409, E12.08889; [38]) and using three different types of impaction samplers (see section 2.2.). The station is located about 30 km west of Copenhagen and immediately east of Roskilde Fjord. The area is dominated by agricultural and recreational usage, but also includes small deciduous forests, especially west of the location.

For the third measurement series, ground-based sampling was performed from the roof of the Danish Meteorological Institute in Copenhagen-Ryparken (N55.71559, E12.56206) at 15 m above sea level, using a 1-day Hirst-type stationary volumetric spore trap [27]. Simultaneously, airborne sampling was done using an aircraft at 457 and 914 m above sea level, while circling above the Institute. The surroundings of the station are of urban character, and the coastline is about 1 km east of the site.

### 2.2 Equipment

Three different samplers were used at Roskilde station.

**Fig. 1:**
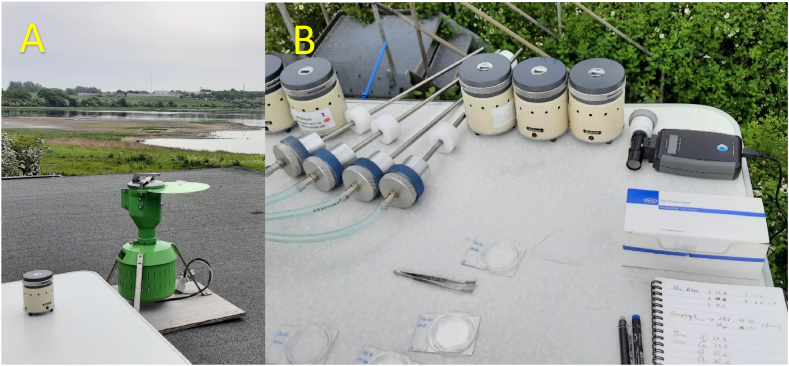
A) A stationary 7- day Hirst-type volumetric sampler and one PVAS placed at the Roskilde station. **B)** 5 PVAS situated next to the 4 isokinetic samplers at Roskilde-Risø. *(Photos by M. Plaza)*.
b) Five Burkard Personal Volumetric Air Samplers – PVAS (Burkard Manufacturing Co. Limited, Rickmansworth, Hertfordshire, England, UK). PVAS are also impactor samplers, but portable and single-stage, with the same principle as the stationary Hirst-type sampler (Fig. 1B). The airflow of 10 l/min is aspirated through a vertically oriented bell-shaped inlet that narrows to an opening of 14 × 1 mm. Particles are deposited on a slide coated with an adhesive medium. Although PVAS was originally designed for the detection of bioparticles indoors, its portability and ease of use has allowed it to be used in several studies also outdoors [28,41].
c) Four specially manufactured impactors (Fig. 2A), shaped for isokinetic sampling from a slow-flying aircraft. The isokinetic impactor (IS) consisted of a 650-mm-long stainless-steel pipe of 4 mm ID, with a sharp inlet facing in the direction of aircraft motion and a 4 mm-ID-jet focussing the air stream onto an impactor surface, bridging a 3-mm gap. The impactor surface was made from interchangeable Isopore polycarbonate membranes (47 mm diameter, 5.0 μm pore size; Merck, Darmstadt, Germany), with the central 10-mm-diameter section sealed using adhesive medium to form a Hirst-type impaction zone opposite to the jet, and the remaining filter surface facilitating the air to pass from the jet and through the filter. The filter was supported by a stainless-steel mesh, with its edge sealed and positioned by an aluminium filter holder (Mesa Laboratories, Lakewood, Colorado, USA). Air was pumped through the impactor at 35 l/min, using an on-board vacuum pump system as described elsewhere [42]. Air flow was measured using a model 4100 flowmeter (TSI, Shoreview, Minnesota, USA). The laminar air flux through the jet calculated at 2.78 × 10^6^ mm/min, corresponding to 46.3 m/s at the impactor inlet, which matches the airspeed of the aircraft.

a) A stationary 7- day Hirst-type [27] volumetric sampler (Burkard Manufacturing Co. Limited, Rickmansworth, Hertfordshire, England, UK) (Fig. 1A). It is considered the reference device for pollen and spore sampling at the European level [12,28,39,40]. Air is aspirated in at a rate of 10 l/min through a 2 × 14 mm slit. Particles enter through the slit and impact on an adhesive tape that moves 2 mm per hour, providing temporally resolved data. The tape is then analysed microscopically, counting the number of pollen grains and fungal spores, and counts are transformed to bioparticles per cubic meter following the recommendations of Galan et al. [29] *and the European standard method (EN 16828:2020).*

**Fig. 2:**
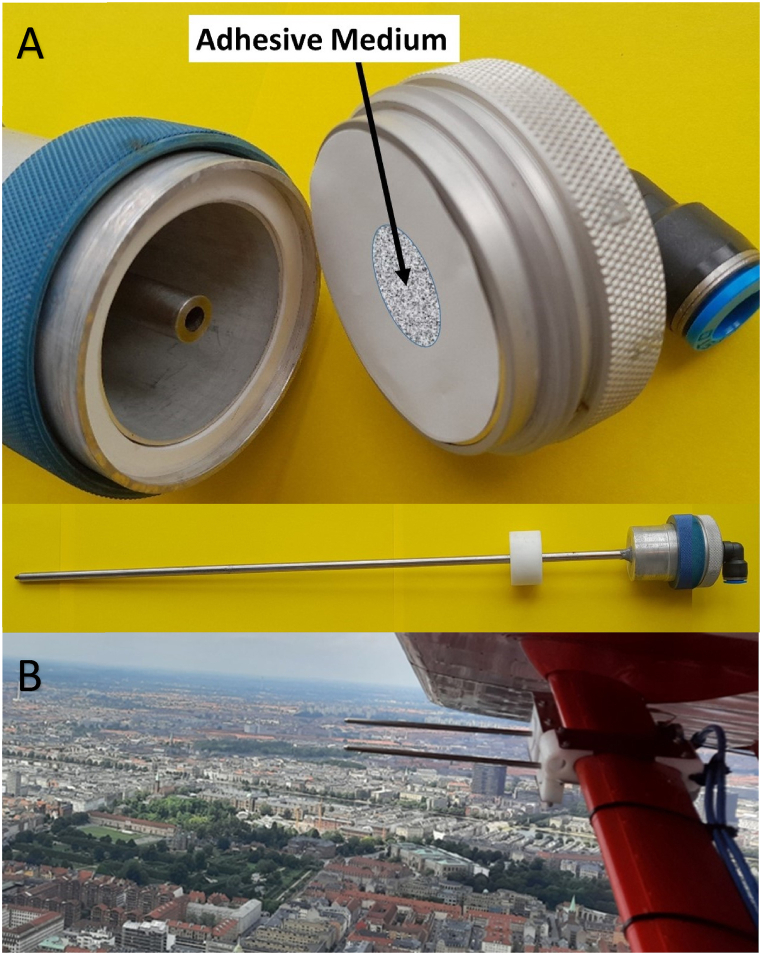
**A.** Details of the isokinetic impactor *(photo by U. Gosewinkel).***B.** Two isokinetic impactors employed from the aircraft flying over Copenhagen *(photo by M.P. Plaza).*

When applied airborne, two isokinetic impactors (IS) were employed from an AP32 ultralight fixed-wing manned aircraft (Fig. 2B) (Aeroprakt, Kiev, Ukraine), modified for aerial surveying. The IS were mounted ≥130 mm under the right wing's lower surface and ≥2180 mm from the aircraft's centerline, i.e. in an area that had been defined by the manufacturer as undisturbed by propeller slipstream and engine exhaust. The inlet tips protruded 125 mm in front, and ≥205 mm below the wing's leading edge, thus remaining free of turbulence generated by the wing. The aircraft type was chosen for its heated cabin, its high inside-cabin payload (2 persons plus 35 kg of instruments), in addition to its legal status facilitating modifications by the owner. Modifications to the airframe were only implemented in places that the manufacturer had defined as structurally non-critical. The aircraft was equipped with the flight instruments required for operating inside controlled airspace. Its maximum permitted altitude was ca. 2,900 m) and the minimum height when overflying residential areas was ca. 300 m. In sampling configuration, i.e. when the vacuum system required additional electrical power from the engine's 24-V alternator, fuel consumption was up to 17 l/h of unleaded gasoline, resulting in a theoretical endurance of 5.3 h on full tanks. Air speed was 167 km/h and the en-route climb rate 12 km/h.

Three different measurement series were done: (1) all devices at ground level at Roskilde-Risø on 7 June 2021; (2) five PVAS, one Hirst-type and two isokinetic impactors at ground level, and simultaneously two isokinetic impactors at 150 m altitude at Roskilde-Risø on 9 June 2021; and (3) two isokinetic impactors at two different heights each (457 m and 914 m) over Copenhagen-Ryparken on 11 June 2021.

### 2.3 Sampling and standardisation

Pollen and spore sampling, and preparation of slides, were performed according to the minimum recommendations by the European Aerobiology Society (EAS) [29] *and the European standard method (EN 16828:2020)*. Pollen was counted at 400× magnification, under an optical microscope. Since the measurements of each device were made during a short period, no more than 30 min, it was decided to identify and count all the pollen and fungal spores collected in the samples.

In order to standardize the pollen and spore measurement technique using an aircraft, the ‘gold-standard’ techniques [27] versus the newly developed isokinetic impactors were applied simultaneously in the field at the Roskilde-Risø station.

All devices operate on the principle of pumping air volumetrically at a specific and stable inflow rate and impacting bioaerosols on a surface coated with adhesive medium. The aircraft sampler performs isokinetically at the aircraft's speed, and volumetrically when not in motion.

Initial ground samples were taken in different timeframes throughout a day, viz. 10 min, 20 min and 30 min at Roskilde-Risø. Airflow for each sampler was assessed frequently using a digital flow meter throughout the sampling. Flow to the Hirst trap was measured using a hot-wire flowmeter as recommended in Ref. [43]. Samples were fixed and stained on glass slides and mounted under glass cover slips. Then, all types of pollen taxa and spores were identified and quantified microscopically. All counts were transformed to particles per m^3^ of air [29,40].

### 2.4 Implementation

Accordingly, for the second and third measurement series, the selected optimal techniques were applied in the field, under different vegetation and urbanisation environments.

For the second measurement series, a sampling flight was carried out at 150 m above Roskilde Fjord, upwind from the Roskilde-Risø station, taking two airborne samples. Ground-based reference samples were taken simultaneously at the ground sampling station, using two duplicate isokinetic impactors, five portable samplers and the stationary 7-day Hirst-type sampler.

For the third sampling series, a sampling flight was made over an urbanised area above the national Danish monitoring site at the Danish Meteorological Institute. Using two isokinetic impactors, two airborne samples were taken above the ground-level station (30 min per sample), one at 457 m and one at 914 m, both altitudes being within the planetary boundary layer.

### 2.5 Data analysis

Patterns of abundance of pollen grains and fungal spores were considered. All data were processed as bioaerosol particle concentrations per cubic metre of air. Statistical analysis was performed for those pollen and spore taxa that contributed more than 0.5 % to the total concentration, respectively, for both ground and aircraft sampling.

## 3 Results

### 3.1 Pollen diversity and concentration at ground level

Results obtained from the 5 portable Burkard samplers (PVAS), the stationary Burkard sampler and 4 isokinetic impactors, all operating simultaneously at 10, 20 and 30 min at Roskilde-Risø are shown in Table S1 in supplementary material.

**Fig. 3:**
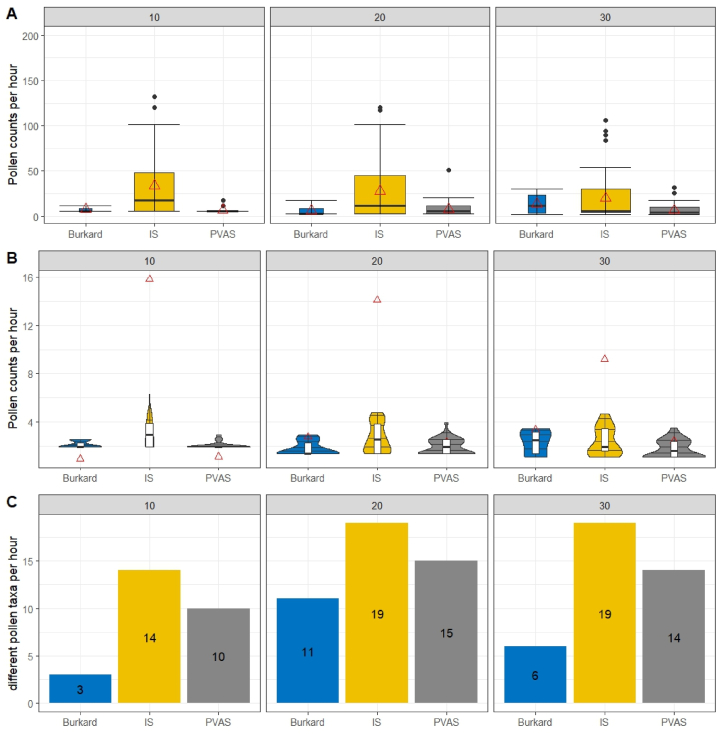
**(A)** Pollen counts per hour with standard error, median (black line) and mean (red triangle) **(B)** Violin plot with pollen counts per hour and quantiles 25, 50, 75 and 95, mean (red triangle) **(C)** biodiversity (number of different plant taxa) with the three different devices used for 10, 20 and 30 min of sampling duration. All samples taken side by side on ground (first measurement series). Abbreviations for sampler types as in Table 1. (For interpretation of the references to colour in this figure legend, the reader is referred to the Web version of this article.)

At ground level, pollen concentration averages (Table 1) were higher with the isokinetic impactor, with approximately 3.5 times higher inflow rate compared to the Burkard samplers. The concentrations obtained in almost all cases (Fig. S1) revealed that measurements with Burkard traps gave consistently lower values, especially for short periods of time (Table 1, Fig. S1). To highlight even more the comparability and efficiency of all devices, we also compared per time interval (pollen counts per hour), so as to eliminate any sensitivity differences over time. It was then even more pronounced that the isokinetic sampler was steadily with the highest abundance and diversity of pollen in five out of the six sampling intervals (Fig. 3A–C, Fig. S1). The best timeframe for the isokinetic impactor was found to be 10 min, with the highest amount recorded in any of the durations sampled.

**Table 1:** Mean, standard error (SE), upper and lower values in each device for logarithmic pollen concentration (grains/m^3^) obtained for 10, 20 and 30 min of sampling duration, using a Hirst-type stationary sampler (“Burkard”), five Burkard Personal Volumetric Air Samplers (“PVAS”) and four isokinetic impactors (“IS”) side by side on ground.

Duration	Device	Mean	SE	Upper	Lower
10	Burkard	1.67	0.98	3.63	−0.30
10	PVAS	2.08	0.70	3.48	0.69
10	IS	7.43	4.25	15.93	−1.07
20	Burkard	4.58	1.62	7.82	1.35
20	PVAS	4.00	1.58	7.16	0.84
20	IS	6.40	2.54	11.49	1.32
30	Burkard	5.65	2.75	11.15	0.14
30	PVAS	3.80	1.46	6.72	0.88
30	IS	4.20	1.89	7.98	0.42

### 3.2 Pollen concentration and diversity at different heights

During the second sampling series, pollen grains were sampled by using the five PVAS along with two isokinetic impactors operating simultaneously at ground level during 20 min, at the same time that two other isokinetic impactors were sampling from the aircraft flying over the same area at 300 m altitude. In the third series, pollen was sampled over Copenhagen using two isokinetic impactors at 457 and 914 m (Table S1).

**Fig. 4:**
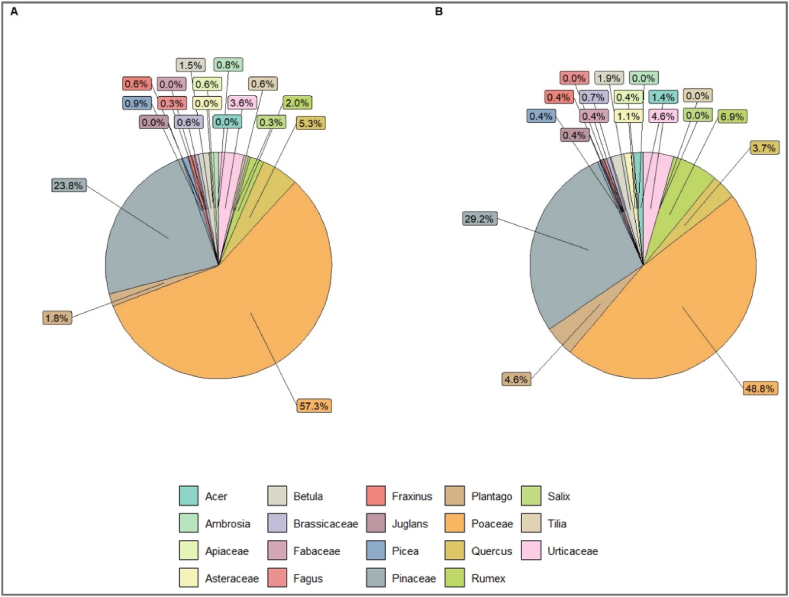
Pollen biodiversity and abundance at both **(A)** ground and **(B)** higher (300 m) altitude, using PVAS and isokinetic impactor at ground level and the isokinetic impactor at higher altitude.

The results showed that airborne pollen biodiversity is not lost with altitude; practically the same taxa were found at ground level (Fig. 4A), 300 m (Fig. 4B) or higher altitudes, 16 pollen taxa are found at both, high and ground level. The percentages in concentrations of these pollen types also remain generally similar, even with the difference in altitude (Fig. 4A–B).

**Fig. 5:**
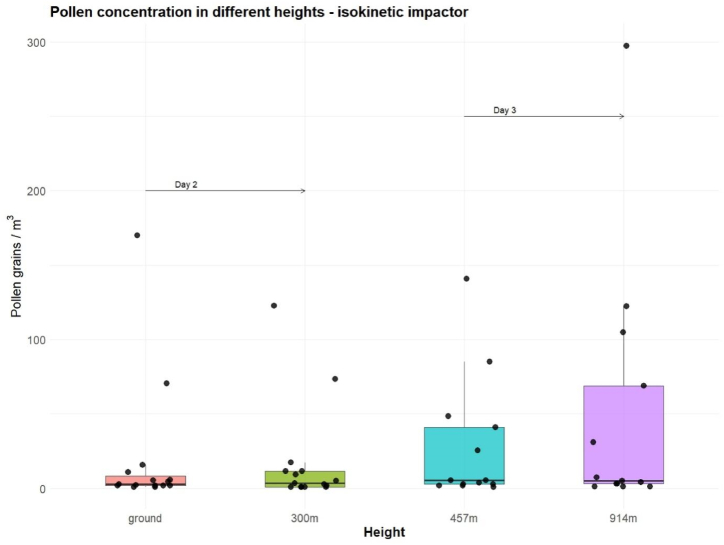
Distribution of pollen concentrations collected with the isokinetic impactor at different heights. Day 2 refers to second, Day 3 to third measurement series.

As to pollen concentration, Fig. 5 depicts a comparison among the concentrations along the range and distribution for the different heights on two different sampling days. We observed a greater variability as well as larger outliers at high altitude. During Day 2 the concentration was slightly higher at 300 m altitude, although the percentage of each of the pollen taxa did not change. During Day 3, the concentrations were higher than on the previous days, and some pollen types were especially abundant, as in the case of *Poaceae*, *Quercus* and *Pinus*. The ground station data showed a similar trend in pollen concentrations during the same day, with high concentrations of *Poaceae*, *Quercus* and *Pinus,* but significantly lower than those collected with the aircraft at 917 m altitude (data not shown).

**Fig. 6:**
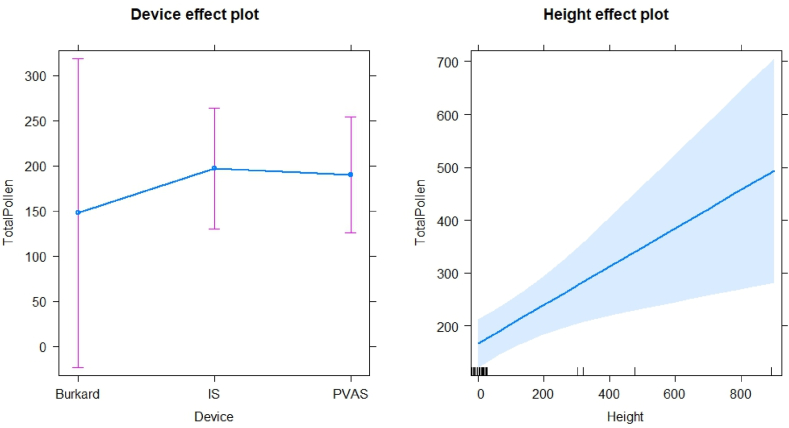
Analysis of two-way ANOVA of total pollen collected with the different devices at ground level, 300 m, 457 m and 914 m altitude.

For pollen, a two-way ANOVA (Fig. 6) was run to determine effects on concentration. There were significant effects of height (ANOVA: F = 14.21, df = 1, p < 0.001), and sampling day (F = 16.48, df = 1, p < 0.001) on the total pollen concentration in the air, whilst the device used was not significant (F = 1.92, df = 2, p > 0.1). Post hoc analysis was performed with Bonferroni adjustment. The mean pollen concentration was statistically significantly lower at ground level (162±18.8) compared to 300 m (258±69.1) and 457 m (650 +/97.7), *p* = 0.006. This indicates that the effect of pollen concentration collected depended on the altitude and the sampling day, but not on the device used.

### 3.3 Fungal spore concentration and diversity at different heights

In order to test the ability of the impactor to sample spores of different sizes and at different altitudes, fungal spores were also quantified microscopically. Thirty fungal spore taxa were detected with the isokinetic impactor at the different heights during the same measurement series as for pollen (Table S2).

**Fig. 7:**
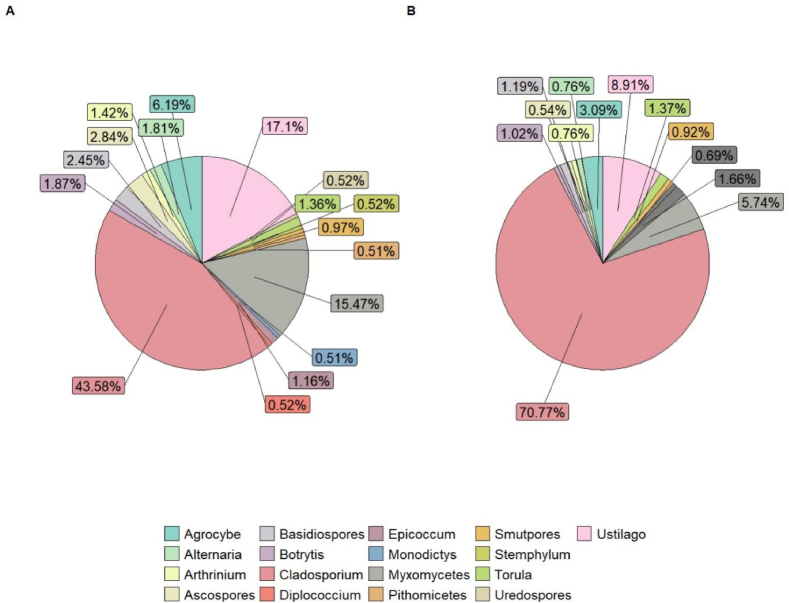
Spore biodiversity and abundance (>0.5) at **(A)** ground and **(B)** higher (300 m) altitude, using two isokinetic impactors at each altitude simultaneously.

Fig. 7A–B shows the differences in concentration at two different heights. At ground level 17 taxa are the most abundant (>0.5 %) and 13 taxa at higher altitude (abundance >0.5 %). In both cases, *Cladosporium* is by far the most abundant taxon, representing more than 70 % in the samples collected at more than 300 m high. On the other hand, some taxa reduce their concentration in high altitude, *Ustilago* and Basidiospores, but others keep their percentage in both heights, i.e. *Torula* and *Pithomyces*. *Alternaria* spore concentration remained low in both heights, probably because the main season had not started yet during the study campaign.

**Fig. 8:**
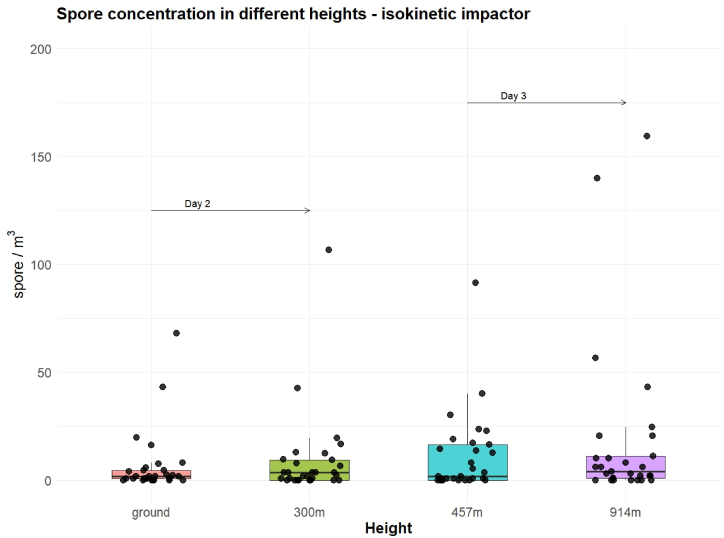
Distribution of fungal spore concentrations collected with the isokinetic impactor at different heights. Day 2 refers to second, Day 3 to third measurement series.

Furthermore, as with the pollen results, Fig. 8 shows a comparison of spore concentrations across the range and distribution for the different altitudes on two different sampling days. We observed a higher variability as well as larger outliers at the high altitude. Nonetheless, we still found that more fungal spores were observed with higher altitude, even within such a small range of heights (Fig. 8). The average concentration was slightly higher at 300 m altitude on day 2. Concentrations were higher at more than 450 m high, and some spore taxa were particularly abundant, such as *Cladosporium*, *Ustilago* and Basidiospores.

## 4 Discussion

The presence of bioaerosols (i.e. pollen grains and fungal spores) is considered to be most evident near the sources, in the lower part of the troposphere, where particle movement is essentially influenced by turbulence and convective currents [44]. The microscopic size of these bioaerosols (<100 μm) [45], facilitates their transport in the atmosphere. On the other hand, certain atmospheric events can affect the diversity of such biological particles, and allow them to travel long distances and also to be detected at unusual times of the year [41]. This seems to be the case for ragweed pollen, which has been detected in our samplers at a time before its main pollination period. That implies that the pollen may have come from a longer-distance source.

In spite of our knowledge about horizontal transportation, the vertical distribution of bioaerosols at high altitudes remains unclear and evidence is still scarce. Recently, some studies have been carried out to understand how the concentration and diversity of pollen grains and/or spores change with altitude, but generally at a maximum of 30 m [13,46], and the differences in abundance between height levels were highly correlated with climatic conditions, especially wind speed and precipitation [47]. In most of the cases where the difference in pollen at different heights was studied, higher pollen concentrations were detected at ground level, but the counts were significantly correlating between heights [13,48]. In contrast, other studies showed similar patterns at two different heights for some taxa (i.e. *Betula*), while Poaceae, Urticaceae or *Artemisia* showed differences in pollen concentrations at different altitudes [49,50]; although these differences are local and do not apply to all locations [46]. On the other hand, Rojo et al. [13] showed that pollen concentrations are much more homogeneous above 10 m above ground level.

All these studies have the added difficulty that buildings and local sources have an important influence [50], in addition to meteorological variables. At higher altitudes, drones, planes or balloons are required. It is still difficult to draw conclusions about how bioaerosol abundances vary with height, because the sampling procedures and analytical techniques are different [47,51]. A previous study carried out in a light aircraft compared with data from a car, recorded similar results for pollen differences at high altitude [20], with higher biodiversity at high altitude, which is consistent with our results. Furthermore, in the aircraft samples, the majority of pollen taxa were more abundant, whereas in the ground level samples the Poaceae pollen was by far the most abundant, which confirms our results. This study [20] yielded similar results to our own here, even though it was implemented about a decade ago and in a completely different setting (Thessaloniki, Greece, Mediterranean climate and lower altitude), and highlighted the possibility but also the frequency of longer-distance bioaerosol transport and the importance of vertical movement of particles. Moreover, in a spore sampling carried out on several manned flights in 1967 [52], most profiles showed maximum concentrations between 600 and 1200 m, which were 2–4 times higher than those in the surface air below, and pollen was found in profiles from 300 to 900 m, which are exactly the altitudes we considered in our study. Comtois et al. [18] used a hot-air balloon and found pollen at 600 m altitude, with a trend towards higher pollen concentrations at higher elevations, as we have observed in most of our results. In the case of grasses, although high concentration has been found at high altitudes, the concentration was higher at ground level, in the same time period.

Regarding spores, Hirst et al. [52] found *Cladosporium* spores up to altitudes of 1,800 m. We flew at a maximum of 900 m, but we also noticed the large concentration of them at high altitude, especially *Cladosporium*. The transported spores are so abundant that, if viable and effectively deposited by rain, even minor components of the cloud could have intense effects on crops. Likewise, Damialis et al. [20] found that the majority of spores from almost all fungal taxa were most abundant at higher altitudes, even at elevation more than 2 km above ground level.

If one adds the variable of vegetation (Fig. S2) into the bioaerosol transport equation, for our study we conclude that the majority of captured pollen taxa (Fig. 4) could actually be represented by the flight measurements, but at the same time it is confirmed that pollen from specific taxa probably originates from longer distances, as in the case of the forest species from *Fagus*, *Picea*, *Quercus*. To the best of our knowledge, species from these taxa are not abundantly present in the region to justify such a high relative abundance of their pollen in our samples.

All measurements so far have been relying on traditional biomonitoring techniques and certain hypotheses. The efficiency of vertically oriented aerosol samplers, such as the Hirst type impactors, is known to vary with particle size, and to decline rapidly as wind speed increases [53]. The latter called for the development of an isokinetic impactor specifically for the airspeed of the aircraft chosen. In its current design, our new device does not provide a time resolved sample. However, a limited temporal resolution can be achieved by switching samples during each flight. On Hirst sampler and PVAS, the impaction surfaces consist of non-porous material, e.g., glass or Melinex tape. To the best of our knowledge, the use of a section of a microporous filter for mounting the adhesive medium on the isokinetic impactor, is a novelty. In the future, this principle may facilitate simultaneous collection of bioaerosols that are too small to be impacted along with pollen, on separate sections of the filter. The need for such devices has surfaced recently, with air samples being subjected to total air biome analysis [54].

The new ultralight-based instrument platform proved versatile with respect to payload, and uncomplicated with respect to instrumentation mounted on the outside of the aircraft. On the other hand, having only two isokinetic impactors available, severely limited the number of samples that we could take during one flight, as change of impaction-filter required to land the aircraft. This could be improved by mounting more isokinetic impactors on the aircraft. We utilized a manned aircraft, as a prerequisite of operating inside controlled airspace, in an area of high traffic density and over a populated area, where the use of unmanned aerial vehicles is greatly restricted. One of our flights was conducted inside the controlled airspace of Copenhagen international airport, within 9 km from the runways. During flight, the instrument platform was successfully operated under air traffic rules applicable to General Aviation, which facilitated a high degree of responsiveness by ATC to *ad hoc* changes of flight plans.

The crew size of two allowed for workload distribution between pilot and instrument operator. Potential drawbacks of the aircraft method include its maximum altitude limit, and its minimum height limit when overflying residential areas. Cost of flight and the specialized training required to fly the plane, could be a drawback, but the advantages that this type of sampling can offer information-wise are noteworthy. Also, because the possibility of flying depends on weather conditions, a bias may arise towards calmer and clearer days, as it will be more difficult to obtain data on days with more turbulent or rainy conditions.

Airborne allergenic pollen and fungal spore sampling has traditionally been based on volumetric samplers, morphological identification and counting through a microscope. The technique described here preserves this technology, used as a ‘gold standard’ in aerobiology, and expands it to include the detection of bioaerosols in high altitudes. So, the big question this work attempts to answer here lies on which is the actual biodiversity and abundance and which is the most appropriate technique to draw a safe conclusion. In this study, we demonstrated that the novel biomonitoring method (isokinetic) seems to be currently the most sensitive, capturing a higher number of taxa of both pollen and spores.

That said, it is evident that our aircraft platform was able to capture biologically meaningful data during this study, Undoubtedly, sampling at these heights and using appropriate sampling regimes can provide us with valuable information that can enable us to understand the vectors of bioaerosol transportation along the vertical and horizontal profile of the atmosphere, even above the boundary layer, where other sampling devices cannot be used. Our study suggests that the hypothesized biodiversity and abundance of bioaerosols, as measured until today, may not be exactly accurate or representative, and the aerobiological scientific community needs to explore further, higher and beyond.

## CRediT authorship contribution statement

**Maria P. Plaza:** Writing – review & editing, Writing – original draft, Visualization, Validation, Methodology, Investigation, Formal analysis, Data curation, Conceptualization. **Athanasios Charalampopoulos:** Writing – review & editing, Writing – original draft, Visualization, Validation, Methodology, Investigation, Formal analysis, Data curation, Conceptualization. **Vivien Leier-Wirtz:** Methodology, Investigation, Data curation. **Pia Viuf Ørby:** Writing – review & editing, Data curation. **Mathilde Kloster:** Writing – review & editing, Data curation. **Michael Dines Christiansen:** Writing – review & editing, Methodology, Conceptualization. **Claudia Traidl-Hoffmann:** Writing – review & editing, Investigation. **Athanasios Damialis:** Writing – review & editing, Visualization, Supervision, Methodology, Investigation, Conceptualization. **Ulrich Gosewinkel:** Writing – review & editing, Supervision, Methodology, Investigation, Conceptualization.

## Declaration of competing interest

The authors declare the following financial interests/personal relationships which may be considered as potential competing interests: Prof. Athanasios Damialis, is the Section Editor in Heliyon Agriculture. If there are other authors, they declare that they have no known competing financial interests or personal relationships that could have appeared to influence the work reported in this paper.
