# Supplementary material for: Optimisation of bioaerosol sampling using an ultralight aircraft: A novel approach in determining the 3-D atmospheric biodiversity
Source: Heliyon. 2024 Oct 12;10(20):e38924. doi: 10.1016/j.heliyon.2024.e38924 (PMC11531622; doi:10.1016/j.heliyon.2024.e38924)
Supplement: Multimedia component 1 [file mmc1.docx]

**SUPPLEMENTARY MATERIAL**

**Table S1. In order to standardize the pollen measurement technique with the new isokinetic device,** pollen concentration was measured using different devices at ground level during 10, 20 and 30 min sampling duration. Abbreviations: Burkard, Hirst-type stationary sampler; PVAS, Burkard Personal Volumetric Air Sampler; IS, isokinetic impactor or aircraft sampler.

| **Date** | 07.06.2021 | | | 07.06.2021 | | | 07.06.2021 | | |
| --- | --- | --- | --- | --- | --- | --- | --- | --- | --- |
| **Start Time** | 15:05 | | | 15:51 | | | 16:23 | | |
| **Duration (min)** | **20** | | | **10** | | | **30** | | |
| **Device** | **Burkard** | **PVAS** | **IS** | **Burkard** | **PVAS** | **IS** | **Burkard** | **PVAS** | **IS** |
| ***Acer*** | 0,00 | 0,40 | 0,25 | 0,00 | 0,00 | 0,25 | 0,00 | 0,40 | 0,00 |
| ***Alnus*** | 0,00 | 0,00 | 0,50 | 0,00 | 0,00 | 0,00 | 0,00 | 0,00 | 0,00 |
| **Apiaceae** | 0,00 | 0,00 | 1,25 | 0,00 | 0,00 | 0,25 | 0,00 | 0,00 | 0,25 |
| ***Artemisia*** | 0,00 | 0,00 | 0,00 | 0,00 | 0,00 | 0,00 | 0,00 | 0,00 | 0,25 |
| **Asteraceae** | 1,00 | 0,20 | 4,00 | 0,00 | 0,00 | 0,00 | 0,00 | 0,20 | 4,75 |
| ***Betula*** | 1,00 | 0,60 | 2,25 | 1,00 | 0,50 | 1,00 | 0,00 | 1,60 | 1,75 |
| **Brassicaceae** | 1,00 | 0,00 | 0,00 | 0,00 | 0,00 | 0,00 | 0,00 | 0,20 | 0,75 |
| ***Carpinus*/*Ostrya*** | 0,00 | 0,20 | 0,00 | 0,00 | 0,25 | 0,00 | 0,00 | 0,00 | 0,00 |
| **Chenopodiaceae** | 1,00 | 0,20 | 3,25 | 0,00 | 0,00 | 1,00 | 0,00 | 1,40 | 3,00 |
| **Cupressaceae** | 1,00 | 0,20 | 1,75 | 0,00 | 0,25 | 0,50 | 1,00 | 0,40 | 2,00 |
| **Cyperaceae** | 0,00 | 0,00 | 0,00 | 0,00 | 0,00 | 0,00 | 0,00 | 0,00 | 0,00 |
| **Ericaceae** | 0,00 | 0,00 | 0,25 | 0,00 | 0,00 | 0,00 | 0,00 | 0,00 | 0,00 |
| **Fabaceae** | 0,00 | 0,00 | 0,00 | 0,00 | 0,00 | 0,25 | 0,00 | 0,00 | 0,00 |
| ***Fraxinus*** | 0,00 | 0,00 | 0,00 | 0,00 | 0,00 | 0,00 | 0,00 | 0,00 | 0,25 |
| ***Juglans*** | 0,00 | 0,20 | 0,75 | 0,00 | 0,00 | 0,00 | 0,00 | 0,00 | 0,00 |
| ***Picea*** | 0,00 | 0,00 | 0,25 | 0,00 | 0,25 | 0,25 | 0,00 | 0,00 | 0,25 |
| **Pinaceae** | 6,00 | 4,80 | 36,50 | 1,00 | 1,00 | 36,25 | 15,00 | 9,00 | 46,75 |
| ***Plantago*** | 2,00 | 2,80 | 18,75 | 0,00 | 0,50 | 3,75 | 4,00 | 4,00 | 11,75 |
| **Poaceae** | 4,00 | 5,60 | 18,75 | 0,00 | 0,50 | 6,50 | 13,00 | 4,80 | 19,75 |
| ***Quercus*** | 4,00 | 2,20 | 21,75 | 2,00 | 1,25 | 13,00 | 8,00 | 5,40 | 17,00 |
| **Rosaceae** | 0,00 | 0,00 | 0,00 | 0,00 | 0,00 | 0,00 | 0,00 | 0,00 | 0,50 |
| ***Rumex*** | 0,00 | 0,00 | 0,75 | 0,00 | 0,00 | 0,25 | 0,00 | 0,40 | 0,25 |
| ***Salix*** | 0,00 | 0,20 | 0,50 | 0,00 | 0,00 | 0,50 | 0,00 | 0,00 | 0,50 |
| **Urticaceae** | 1,00 | 1,60 | 3,75 | 0,00 | 1,00 | 2,25 | 1,00 | 1,40 | 4,25 |
| **Unidentified** | 1,00 | 0,40 | 1,00 | 0,00 | 0,25 | 0,00 | 0,00 | 1,00 | 0,75 |
| **Total Pollen/m^3^** | **112,75** | **96,59** | **155,81** | **39,22** | **54,45** | **180,78** | **135,92** | **94,01** | **102,02** |

**Table S2.** Fungal spore concentration measured by isokinetic impactor at different altitudes (ground, 300m, 457m and 914m) during 30 minutes sampling.

| **Date** | **09.06.2021** | **09.06.2021** | **09.06.2021** | **09.06.2021** | **11.06.2021** | **11.06.2021** |
| --- | --- | --- | --- | --- | --- | --- |
| **Level** | ***ground*** | ***ground*** | ***300 m*** | ***300 m*** | ***457m*** | ***914m*** |
| ***Acrodictys*** |  |  |  |  |  | 2 |
| ***Agrocybe*** | 56 | 40 | 30 | 66 | 26 | 55 |
| ***Alternaria*** | 17 | 11 | 9 | 4 | 18 | 11 |
| ***Arthrinium*** | 13 | 9 | 7 | 10 | 19 | 6 |
| **Ascospores** | 28 | 16 | 3 | 6 | 16 | 4 |
| **Basidiospores** | 21 | 17 | 12 | 24 | 21 | 10 |
| ***Botrytis*** | 19 | 10 | 7 | 11 | 15 | 24 |
| ***Cercospora*** | 1 |  |  | 1 |  |  |
| ***Cladosporium*** | 361 | 316 | 341 | 418 | 1718 | 1395 |
| ***Diplococcium*** | 4 |  |  | 2 |  | 1 |
| ***Drechslera*-type** | 1 | 3 |  |  | 2 | 2 |
| ***Epicoccum*** | 9 | 9 |  | 1 | 2 | 6 |
| ***Exosporium*** | 2 |  |  |  | 6 | 3 |
| ***Fusariella*** |  | 1 |  |  |  |  |
| ***Fusicladium*** |  | 1 | 3 | 3 |  | 10 |
| ***Ganoderma*** |  |  |  | 2 |  |  |
| ***Leptosphaeria*** | 2 | 3 | 4 | 1 | 4 | 3 |
| ***Monodictys*** |  | 4 |  | 1 |  |  |
| **Myxomycetes** | 140 | 100 | 109 | 42 | 25 | 155 |
| ***Periconia*** |  |  | 1 | 3 | 44 | 42 |
| ***Peronospora*** |  |  | 2 | 8 |  | 20 |
| ***Pithomyces*** | 2 | 6 |  | 2 | 1 |  |
| ***Polytrichium*** |  |  |  | 1 | 1 | 1 |
| ***Pleospora*** |  |  |  | 1 | 1 | 2 |
| **Smut spores** | 9 | 6 | 7 | 3 | 33 | 6 |
| ***Stemphylium*** | 4 |  |  |  | 1 |  |
| ***Tetraploa*** |  |  |  |  | 1 |  |
| ***Torula*** | 16 | 5 | 28 | 16 | 14 | 20 |
| **Uredospores** | 4 |  | 1 | 1 | 9 | 8 |
| ***Ustilago*** | 124 | 142 | 147 | 124 | 100 | 136 |
| **TOTAL counts** | 833 | 699 | 711 | 751 | 2077 | 1922 |
| **spores/m^3^** | 917,133 | 788,472 | 799,875 | 844,875 | 2018,2209 | 2104,59 |


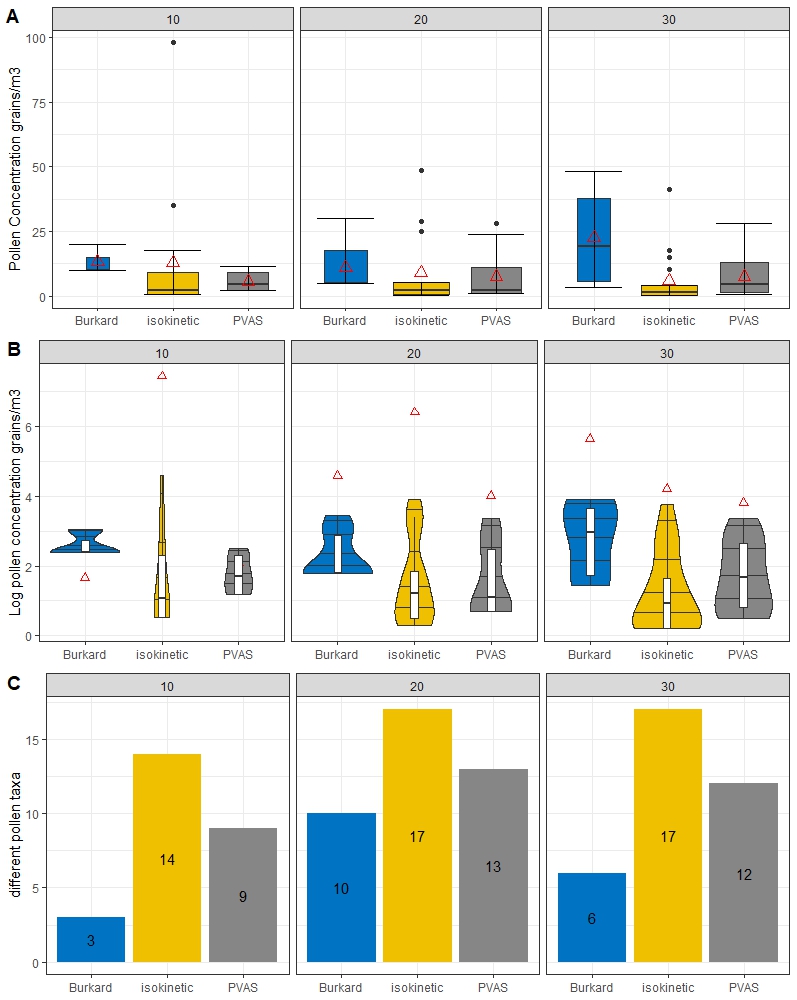


**Fig. S1**. **(A)** Pollen concentration (grains/m^3^) with standard error, median (black line) and mean (red triangle) **(B)** Violin plot with logarithmic pollen concentration (grains/m^3^) and quantiles 25, 50, 75 and 95, mean (red triangle) **(C)** biodiversity (number of different plant taxa) at ground level with the three different devices used for 10, 20 and 30 min of sampling duration. All samples taken side by side on ground (first measurement series). Abbreviations for sampler types as in Table 1.


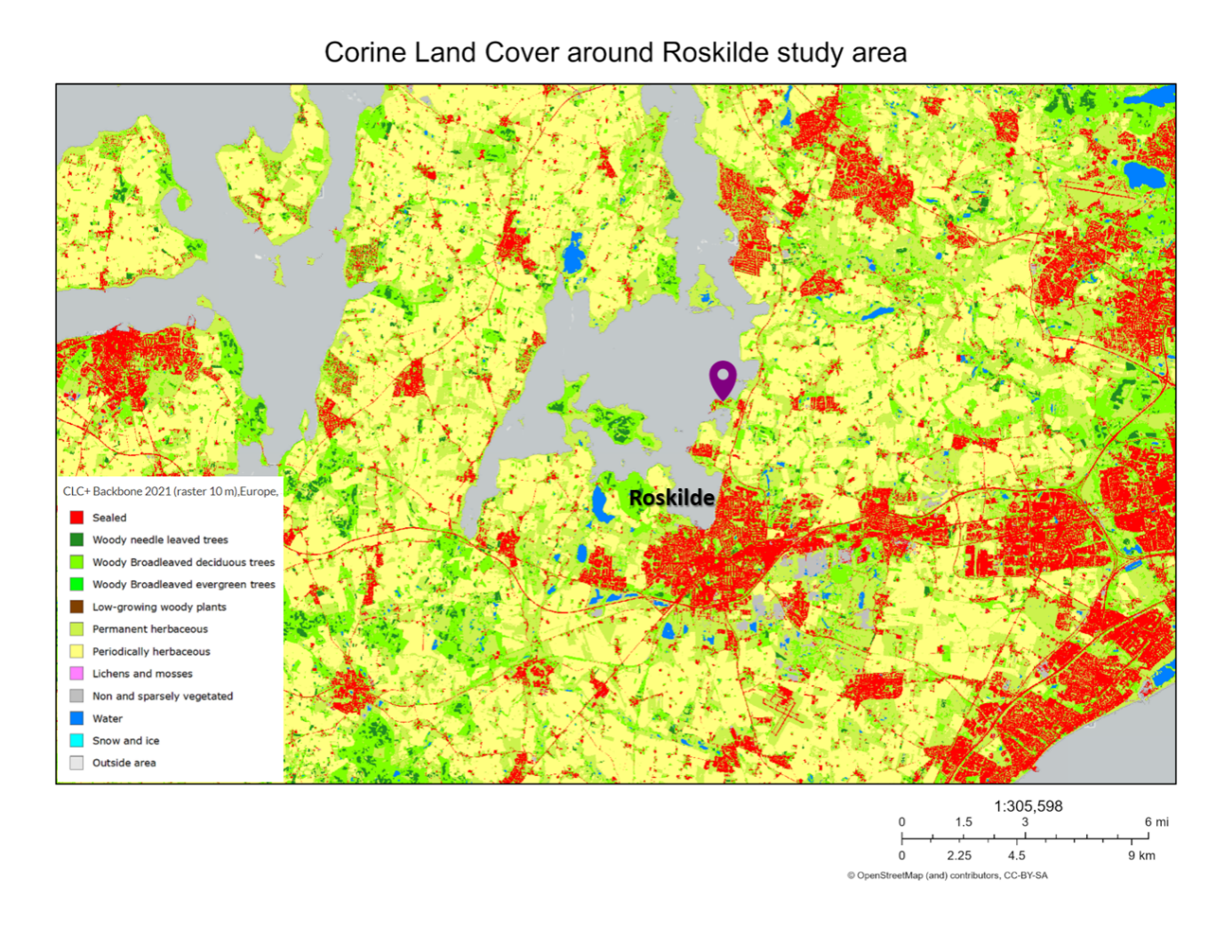


**Fig. S2**. Land use map (Corine Land Cover Classes for the 2021 reference year, each pixel showing the dominant land cover among the 11 basic land cover classes as 10 m raster) of the Roskilde-Risø study area within a radius of approx. 15 km.
